# Bacterial Dormancy Is More Prevalent in Freshwater than Hypersaline Lakes

**DOI:** 10.3389/fmicb.2016.00853

**Published:** 2016-06-09

**Authors:** Zachary T. Aanderud, Joshua C. Vert, Jay T. Lennon, Tylan W. Magnusson, Donald P. Breakwell, Alan R. Harker

**Affiliations:** ^1^Department of Plant and Wildlife Sciences, Brigham Young UniversityProvo, UT, USA; ^2^Department of Microbiology and Molecular Biology, Brigham Young UniversityProvo, UT, USA; ^3^Department of Biology, Indiana UniversityBloomington, IN, USA

**Keywords:** extremophiles, Great Salt Lake, phosphorus, salinity, seed banks

## Abstract

Bacteria employ a diverse array of strategies to survive under extreme environmental conditions but maintaining these adaptations comes at an energetic cost. If energy reserves drop too low, extremophiles may enter a dormant state to persist. We estimated bacterial dormancy and identified the environmental variables influencing our activity proxy in 10 hypersaline and freshwater lakes across the Western United States. Using ribosomal RNA:DNA ratios as an indicator for bacterial activity, we found that the proportion of the community exhibiting dormancy was 16% lower in hypersaline than freshwater lakes. Based on our indicator variable multiple regression results, saltier conditions in both freshwater and hypersaline lakes increased activity, suggesting that salinity was a robust environmental filter structuring bacterial activity in lake ecosystems. To a lesser degree, higher total phosphorus concentrations reduced dormancy in all lakes. Thus, even under extreme conditions, the competition for resources exerted pressure on activity. Within the compositionally distinct and less diverse hypersaline communities, abundant taxa were disproportionately active and localized in families Microbacteriaceae (Actinobacteria), Nitriliruptoraceae (Actinobacteria), and Rhodobacteraceae (Alphaproteobacteria). Our results are consistent with the view that hypersaline communities are able to capitalize on a seemingly more extreme, yet highly selective, set of conditions and finds that extremophiles may need dormancy less often to thrive and survive.

## Introduction

Bacteria in extreme environments survive and often thrive in environmental conditions that are outside the range experienced by the majority of life (Wardle et al., 2004). Extremotolerant and extremophilic bacteria, which are found in virtually all harsh environments, have motivated a wide range of research including the metabolic functions that have contributed to the evolution of Earth’s biosphere (Javaux, 2006; Pikuta et al., 2007); novel enzymes for biotechnological applications in chemical, food, pharmaceutical industries (van den Burg, 2003; Ferrer et al., 2007); and astrobiological clues for discovering life elsewhere in the universe (Rothschild and Mancinelli, 2001). In addition, extremophiles provide insight into the physiological adaptations and functional traits that affect microbial performance along environmental gradients (Feller and Gerday, 1997; Nealson and Conrad, 1999; Pakchung et al., 2006). For example, extremotolerant and extremophilic bacteria have evolved a diverse array of resistance mechanisms, such as the upregulation of organic osmolytes to deal with hypersalinity (Detkova and Boltyanskaya, 2007), heat-shock proteins to combat high temperatures (Solow and Somkuti, 2000; Pakchung et al., 2006), and antifreezes to survive in subzero conditions (D’Amico et al., 2006; Struvay and Feller, 2012). However, all of these adaptations come at an energetic cost, and if environmental conditions cause energy reserves to drop too low, extremophiles may need to rely on other strategies to ensure survival.

One mechanism, dormancy, is frequently offered as a plausible explanation for the persistence of bacterial populations under suboptimal or harsh conditions (Stevenson, 1978; Nicholson et al., 2000; Dworkin and Shah, 2010; Lennon and Jones, 2011). As a bet-hedging strategy, dormancy builds “seed banks” or reservoirs of inactive individuals that may resuscitated in the future under a different set of conditions (Lennon and Jones, 2011). This mechanism not only protects taxa from extinction (Kalisz and McPeek, 1992; Honnay et al., 2008), alters species interactions (Chesson and Warner, 1981), and influences ecosystem processes (Aanderud et al., 2015), but is prolific, with >90% of biomass and >50% of all bacterial taxa residing in a state of inactivity at any time (Alvarez et al., 1998; Lennon and Jones, 2011; Wang et al., 2014). But the empirical evidence for this mechanism is lacking under the harshest conditions—extreme environments. In environments at the margins of life, the activity of extremophiles and extremotolerant bacteria is sensitive to abiotic factors with many microorganisms becoming metabolically active only when a specific set of environmental conditions are met (Pikuta et al., 2007; Zeldovich et al., 2007; Canganella and Wiegel, 2011). However, extremophiles may not just *tolerate* certain aspects of their extreme environment but also actually *require* it for optimal growth and metabolism (Madigan and Marrs, 1997; Harrison et al., 2013). Thus, rendering specific extreme conditions completely normal for highly adapted extremophiles and potentially allowing them to thrive and employ dormancy less often to survive. Given this perspective, one might not expect dormancy to be a prevalent life history strategy in extreme environments.

Hypersaline lakes and their seemingly more benign analogs, freshwater lakes, not only offer an ideal setting to identify the extent extremophiles employ dormancy but also the abiotic cues structuring bacterial activity. There is evidence that halophilic organisms are capable of using dormancy as a way of contending with hypersalinity and the osmotic stress that it induces. For example, an experimental reduction of hypersaline conditions in lagoon water allowed previously undetected protozoa species to emerge from seed banks (Esteban and Finlay, 2003). Also, viable haloarchaea were isolated from ancient halite evaporite formations millions of years old (Jaakkola et al., 2016). Many bacterial taxa in hypersaline are high specialized to thrive under specific salt concentrations and nutrient levels with narrow overlap existing between specific conditions and species (Rodriguez-Valera et al., 1981; Oren, 2008). But with the high energetic costs of maintaining resistance strategies to combat osmotic stress (Oren, 1999), halophiles and halotolerant bacteria may rely on dormancy to survive and persist. In freshwater lakes, other enviormental cues are thougth to regulate transitions into and out of dormancy. In particular, phosphorus availability influences both bacterial activity (Schindler, 1978; Cole et al., 1993; Jones et al., 1998) and dormancy (Jones and Lennon, 2010). Therefore, under periods of nutrient limitation bacteria may decrease their metabolic activity to avoid competition, starvation, and potentially death (Jones and Lennon, 2010).

In this study, we tested whether bacterial dormancy was more prevalent in extreme hypersaline than freshwater environments and identified the differences in lake chemistry that influenced activity in five freshwater and five hypersaline lakes across the Western United States. We estimated the dormancy of individual taxa from the recovery of 16S rRNA genes and transcripts, based on the assumption that 16S rRNA genes are proxy of which bacteria are present in a sample, while 16S rRNA transcripts provide insight into taxa that are metabolically active (Jones and Lennon, 2010). We employed two different measurements of dormancy: the percentage of bacterial taxa exhibiting dormancy in each lake and the total relative recovery represented by these dormant taxa within the community. We related our dormancy metrics to a suite of chemical characteristics including dissolved O_2_, pH, salinity, total nitrogen (TN), and total phosphorus (TP), and temperature.

## Materials and Methods

### Lakes and Water Chemistry

We sampled water from five hypersaline and five freshwater lakes located in seven states (i.e., AZ, CA, CO, ID, OR, UT, and WA) across the Western United States in the early summer (17 May–23 June 2012). We selected hypersaline lakes based on salinity (≥3.5%) and freshwater lakes that were comparable to at least one of the hypersaline lakes in terms of mean depth. The hypersaline lakes included: Great Salt Lake, North Arm (UT); Great Salt Lake, South Arm (UT); Salton Sea (CA); Abert Lake (OR); Mono Lake (CA); and the freshwater lakes included: Mormon Lake (ID); Riffe Lake (WA); Arivaca Lake (AZ); Lily Lake (CO); and Silverwood Lake (CA). Supplementary Table S1 provides additional information on the elevation, surface area, mean depth, and location of the lakes. The North and South Arm of the Great Salt Lake are considered separate lakes. In 1959, the Union Pacific Railroad constructed a rock-filled causeway: separating the lakes; limiting mixing between them; and causing 95% of the incoming streamflow to enter the South Arm (White et al., 2015). We measured electrical conductivity, dissolved oxygen (O_2_), and temperature *in situ* with an OAKTON EcoTestr EC Low Meter (Oakton Instruments Inc., Vernon Hills, IL, USA) and YSI EcoSense DO 200 meter (YSI Inc., Yellow Springs, OH, USA). Waters were analyzed 1.0 m below the lake surface approximately 200 m from the shoreline. At the same location and two other locations, we collected a composite water sample, consisting of three 1 L subsamples. A portion of the composite sample was placed on ice and transported back to the laboratory for further chemical analyses. We measured salinity with a conductivity bridge (Beckman, Brea, CA, USA) and pH with a Thermo Orion Model 410 pH meter (Thermo Scientific, Beverly, MA, USA). TN was measured by oxidation and subsequent chemiluminescence using a Shimadzu TOC-V equipped with a TNM-1 unit (Shimadzu, Kyoto, Japan). We measured TP by persulfate oxidation of organic phosphorus to phosphate followed by colorimetric analysis (Koroleff, 1983). We tested for differences between hypersaline and freshwater lake chemistry using multiple *t*-tests and a Benjamini–Hochberg correction to control for the false discovery rate associated with multiple comparisons (Benjamini and Hochberg, 1995). Last, in the field, we collected bacterial biomass for molecular analyses from 2 L of the composite water sample on 142 mm 0.2 μm filters (Supor^®^ PES membrane, Pall Life Sciences, Port Washington, NY, USA) using a pressure filtration system (Advantec MFS Inc., Tokyo, Japan). Filters were immediately flash frozen with liquid nitrogen and stored at -80°C.

### rDNA and rRNA Bacterial Communities

We characterized lake bacterial communities using RNA- and DNA-based approaches to make inferences about the activity of bacterial taxa. Because ribosomal RNA has a relatively short half-life and is required for protein synthesis (Flärdh et al., 1992; Bernstein et al., 2002; Steglich et al., 2010), we assumed that bacteria identified from RNA transcripts were metabolically active, while the bacteria recovered from 16S rRNA genes reflect the taxa with varying levels of activity, including organisms that are slow growing and/or dormant (Campbell and Kirchman, 2013; Hugoni et al., 2013). For the remainder of the paper, we refer to communities based on the 16S rRNA gene as “rDNA” and 16S rRNA transcripts as “rRNA.” Nucleic acids were extracted from filters using a PowerSoil DNA Isolation Kit and a RNA PowerSoil Total RNA Isolation Kit (MoBio Corporation, Carlsbad, CA, USA). We reverse transcribed RNA transcripts to cDNA using a SuperScript III, one-step RT-PCR kit (Invitrogen Corporation, Carlsbad, CA, USA). We PCR amplified the V3–V4 region of the16S rRNA gene and cDNA using bacterial specific primer set 515F and 806R with unique 12-nt error-correcting Golay barcodes (Aanderud and Lennon, 2011). The thermal cycle conditions were: an initial denaturation step at 94°C for 3 min followed by 35 cycles of denaturation at 94°C for 45 s, annealing at 50°C for 30 s, and an extension at 72°C for 90 s. After purifying (Agencourt AMPure XP PCR Purification Beckman Coulter Inc., Brea, CA, USA) and pooling PCR amplicons at approximately equimolar concentrations, samples were sequenced at the Brigham Young University DNA Sequencing Center^1^ using a 454 Life Sciences genome sequence FLX (Roche, Branford, CT, USA). We analyzed all sequences using mothur (v. 1.29.2), an open-source, expandable software pipeline for microbial community analysis (Schloss et al., 2009). After removing barcodes and primers, we screened sequences to remove short reads, chimeras, and non-bacterial sequences. First, we eliminated sequences < 250 bp in length and sequences with homopolymers longer than 8 bp. Second, we denoised the sequences with AmpliconNoise (Quince et al., 2011). Third, we removed chimeras using UCHIME (Edgar et al., 2011) and eliminated chloroplast, mitochondria, archaeal, and eukaryotic 16S rRNA gene sequences based on reference sequences from the Ribosomal Database Project (Cole et al., 2009). We then aligned sequences against the SILVA database (Pruesse et al., 2007) with the SEED aligner, created operational taxonomic units (OTUs) based on uncorrected pairwise distances at 97% sequence similarity, and determined the phylogenetic identity of OTUs with the SILVA database.

To characterize variability in bacterial community composition among lakes, first, we used principal coordinates analysis (PCoA) and permutational multivariate analyses of variance (PERMANOVA, Anderson, 2001). The PCoA was based on a Bray–Curtis distance matrix using the ‘vegan’ package in R (R Development Core Team, 2015). While the PCoA aided in the visualization of communities, we tested for the main effects and interactions between lake type (hypersaline vs. freshwater) and nucleotide type (rDNA and rRNA) with PERMANOVA using the *adonis* function also in the ‘vegan’ package of R. Second, we quantified the alpha diversity of communities as the inverse Simpson index (Haegeman et al., 2013) after rarefaction by a common sequence number (5,846) to remove any bias due to differences in sequencing depth among samples (Nipperess and Matsen, 2013). We examined differences in alpha diversity between lake (hypersaline vs. freshwater) and nucleotide (rDNA vs. rRNA) type using two-way ANOVA with a Tukey’s HSD test. Third, we calculated the relative recovery of eleven phyla and four subclasses in rDNA communities to identify differences in the distribution of major taxonomical groups (recovery ≥ 1.0%) between hypersaline and freshwater lakes. Taxonomic trends were shown with a heat map with hierarchal clustering using the *heatmap* function in the ‘gplot’ package in R (Oksanen et al., 2013). Lastly, to evaluate if hypersaline and freshwater environments supported similar numbers of bacteria, we estimated abundance as the number of 16S rRNA gene copies in lakes using quantitative PCR and the bacterial specific primer set 515F and 806R (Aanderud and Lennon, 2011). We tested for differences between hypersaline and freshwater lakes using a *t*-test.

### Bacterial Dormancy Estimates

We used rRNA: rDNA ratios as a proxy to estimate if a given taxa was dormant or active (Jones and Lennon, 2010; Franklin et al., 2013). Specifically, in each lake, we estimated the dormancy of individual OTUs as 1 - (rRNA recovery/rDNA recovery). From each of the resulting values, we classified OTUs as either dormant or active based on a cutoff. The classification of dormant vs. active OTUs is sensitive to the specific cutoff selected (Franklin et al., 2013). Therfore, we estimated dormancy across a range of cutoffs from 0.1 to 0.9. From each of these cutoffs, we estimated bacterial dormancy as the percentage of dormant OTUs occurring in each lake and as the total relative recovery represented by these dormant taxa within the community. Specifically, the percentage of dormant OTUs was calculated as the number of dormant OTUs divided by the total OTUs present in a given lake × 100, while the relative recovery of dormant OTUs was calculated as the sum of all dormant OTUs in each lake. To determine if dormancy was more prevalent in hypersaline environments, we used an indicator variable in multiple regression where lake type (hypersaline vs. freshwater) was treated as a categorical predictor variable. Differences in the slopes or intercepts between lake type suggest that hypersalinity differentially affected dormancy responses across the cutoffs (Lennon and Pfaff, 2005).

### Environmental Drivers of Bacterial Dormancy

We identified the lake chemical characteristics that influenced bacterial dormancy in hypersaline and freshwater lakes using multiple regression with lake as a categorical predictor variable (Neter et al., 1996; Lennon and Pfaff, 2005; Lennon et al., 2013). We tested whether or not a variable (i.e., dissolved oxygen, pH, salinity, TN, and TP) related to the percentage of dormant bacteria occurring in the five hypersaline and five freshwater lakes and the recovery of dormant taxa using forward selection procedure and Akaike’s information criterion (AIC; Akaike, 1998). For these analyses, we used the median cutoff value to classify OTUs as either dormant (≥0.5) or active (<0.5). At this cutoff ratio, the total recovery of an OTU (active and inactive cells) was at least double the recovery of RNA transcripts being produced. Therefore, we assumed that in dormant OTUs no more than half of the bacteria were metabolically active and producing RNA transcripts. In indicator multiple regression, lake chemistry variables were treated as continuous predictor variables and lake type (hypersaline vs. freshwater) was treated as a categorical predictor variable. Differences in the slopes or intercepts between lake type suggest that hypersalinity differentially affected dormancy responses to the chemistry variables. The chemical characteristics were checked for collinearity using the *vif* function in the ‘car’ package in R.

### Rare and Abundance Bacteria and Dormancy

We classified dormant and active OTUs into abundance categories to gain insight into the contribution of rare and abundant taxa to bacterial dormancy. In our study, rare OTUs were defined as OTUs with a relative recovery ≤ 0.1% and all other OTUs were considered abundant with a relative recovery > 0.1% in rDNA communities. Justification for this is based on rank abundance curves of bacterial communities from sequencing efforts. In these curves, the bacterial recovery of 0.1% often represents a visible demarcation between the few abundant OTUs with relatively high recoveries and the thousands of rare OTUs with relatively low recoveries (Pedrós-Alió, 2012). Similar to indicator variables multiple regression analyses, OTUs were classified as either dormant (≥0.5) or active (<0.5). We tested for the effects of lake type (hypersaline vs. freshwater) and activity (dormant vs. active) on the percentage and recovery of rare and abundant OTUs in communities using two-way ANOVA and Tukey’s HSD tests. Further, to evaluate whether dormancy was restricted to specific OTUs, we estimated the number and recovery of dormant rare and abundant OTUs in 45 bacterial families. Similar to dormancy in lakes, we estimated dormancy in families as the percentage of dormant OTUs occurring in a given family in each lake and summed the relative recovery of dormant OTUs for these taxonomical groups. Differences in dormancy among taxonomical groups and lakes were shown in heat maps with hierarchal clustering using the *heatmap* function in the ‘gplot’ package in R (Oksanen et al., 2013).

## Results

### Water Chemistry

Salinity clearly distinguished the extreme conditions in hypersaline lakes from the more benign environmental conditions in freshwater lakes, but other chemical variables also differed between lake types (**Table 1**). On average, salinity was 24-times higher in hypersaline than in freshwater lakes. All hypersaline lakes met the classification of hypersalinity (>3.5%) at the time of sampling, except the Salton Sea (3.0%, Supplementary Table S2). The Salton Sea is often classified as a hypersaline lake, but salinity levels may vary spatially (Hawley et al., 2014). For the purpose of our study, we included the Salton Sea in our hypersaline designation since the salt levels were on the borderline between brackish (0.05–3.0%) and saline (3.0–5.0%). In addition, electrical conductivity was nineteen-times higher in hypersaline lakes, and pH was 8.7 ± 0.47 in hypersaline and 7.0 ± 0.18 in freshwater lakes (mean ± SEM). Conversely, O_2_ levels were 23% lower in hypersaline than freshwater lakes. Based on concentrations of TN and TP, the trophic status of freshwater and hypersaline lakes varied widely from oligotrophic to hypereutrophic, resulting in no differences in total resources between lake types (Vollenweider and Kerekes, 1980; Bachmann et al., 2013).

**Table 1:** Chemistry in freshwater and hypersaline lakes.

	Freshwater	Hypersaline	*P*-value
Dissolved O_2_ (μmol L^-1^)	233 ± 14.93	174 ± 9.005	0.01
Electrical conductivity (dS m^-1^)	4.5 ± 2.52	85 ± 19.1	0.01
pH	7.0 ± 0.175	8.7 ± 0.473	0.02
Salinity (%)	0.29 ± 0.167	7.2 ± 2.07	0.03
Temperature (°C)	18 ± 1.40	21 ± 2.12	0.36
Total N (μmol L^-1^)	30.6 ± 8.992	125 ± 57.78	0.18
Total P (μmol L^-1^)	6.6 ± 5.40	70 ± 33.3	0.14

*Data are mean ± SEM (n = 5) between freshwater and hypersaline lakes with significant differences based on t-tests and a Benjamini–Hochberg adjustment for multiple comparisons (P < 0.05), which resulted in no false discoveries among significant variables.*

### Bacterial Communities in Hypersaline and Freshwater Lakes

**FIGURE 1:**
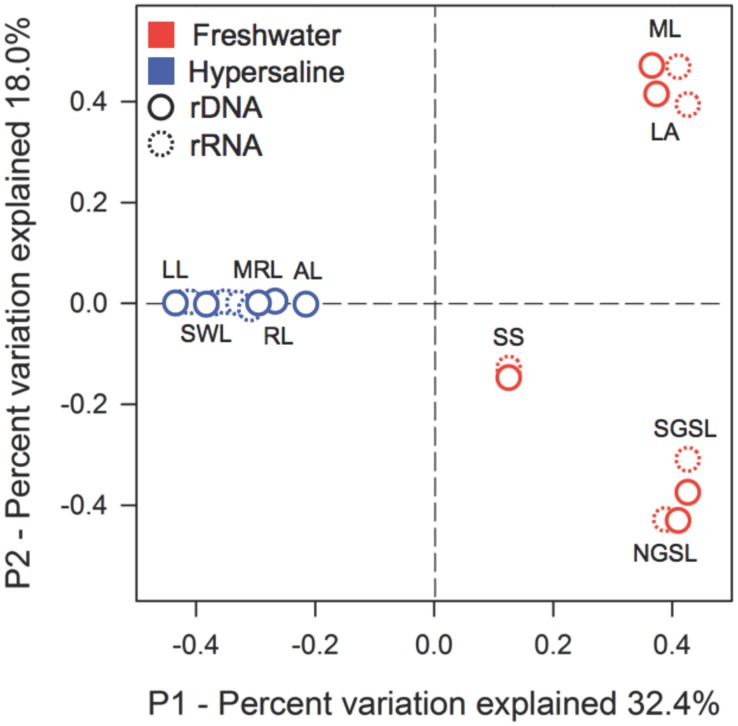
**Extreme hypersaline lakes influenced the composition of active and total bacterial communities.** The multivariate ordination was generated using principle coordinate analysis (PCoA) on a sample × OTU matrix of rDNA and rRNA (indicated by dashed lines) community libraries (97% similarity cutoff). Lake abbreviations are as follows: hypersaline lakes—Great Salt Lake, North Arm (NGSL); Great Salt Lake, South Arm (SGSL); Salton Sea (SS); Abert Lake (LA); Mono Lake (ML); and freshwater lakes—Mormon Lake (MRL); Riffe Lake (RL); Arivaca Lake (AL); Lily Lake (LL); and Silverwood Lake (SWL).

Hypersaline environments had strong effects on the composition of active and total bacterial communities. This inference was based on the recovery of 294,999 quality sequences and 7,430 unique OTUs with samples possessing an average sequencing coverage of 97% ± 0.01. The PCoA results distinctly separated hypersaline from freshwater bacterial communities in ordination space along PCoA axis 1, which explained 32.4% of the variation (**Figure 1**). Hypersaline communities were further separated along PCoA axis 2, which explained 18% of the variation. The PERMANOVA results supported the ordination demonstrating a compositional difference between hypersaline and freshwater communities (PERMANOVA, lake type, *F* = 5.33, *P* = 0.005, df = 1), and also revealed a significant difference between active and total bacterial communities (PERMANOVA, nucleotide type, *F* = 1.9, *P* = 0.03, df = 1).

**FIGURE 2:**
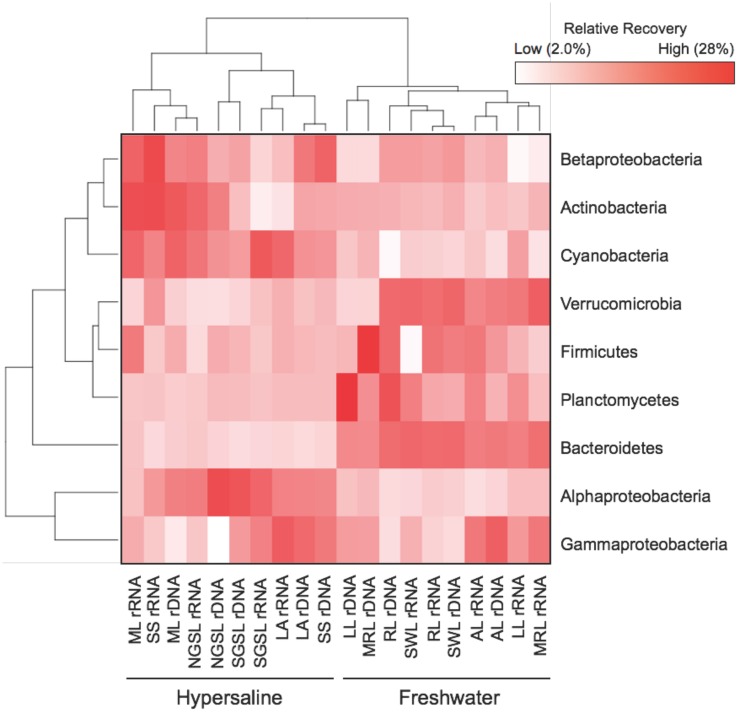
**Heat map showing the distribution of six phyla and three Proteobacteria subclasses that contributed ≥1% of the relative recovery to rDNA and rRNA lake communities.** Values are based on means (*n* = 5) with hierarchal clustering of ecosystem (bottom) and phylum (left).

Despite having similar bacterial densities as freshwater communities, hypersaline communities were less diverse and compositionally similar. Specifically, bacterial diversity was 58% lower in hypersaline than freshwater rDNA communities (two-way ANOVA, lake × nucleotide type, *F* = 15.1, *P* = 0.001, df = 1, Supplementary Figure S1). The distribution of six phyla and three Proteobacteria subclasses distinguished hypersaline from freshwater communities; while rDNA and rRNA communities closely grouped together only within hypersaline lake (**Figure 2**). The recovery of Alphaproteobacteria and Cyanobacteria was at least 2.5- and 1.7-times higher in hypersaline than freshwater rDNA and rRNA communities, respectively. Alternatively, the recovery of Bacteroidetes was 7.1-times lower in hypersaline rDNA and rRNA communities. Based on qPCR of rDNA, hypersaline (5.8 × 10^6^ ± 4.41 × 10^6^ copies 16S rDNA L^-1^ water) and freshwater lakes (1.1 × 10^7^ ± 1.00 × 10^7^ copies 16S rDNA L^-1^ water) bacterial densities were comparable (*t*-test, *t* = 0.24, *P* = 0.64, df = 1).

### Bacterial Dormancy Estimates in Lakes

**FIGURE 3:**
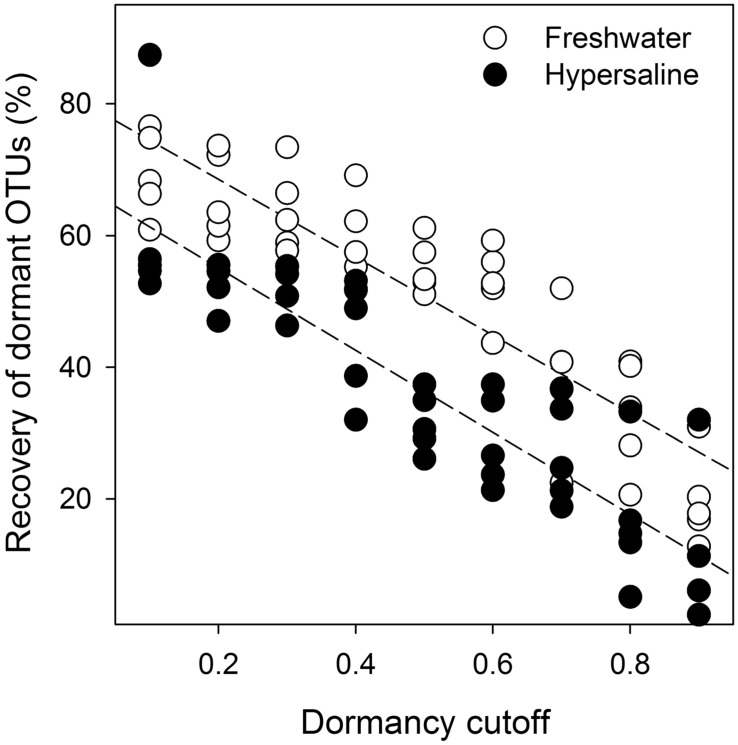
**Bacterial dormancy decreased linearly as the cutoffs estimating dormancy increased or became more stringent and was more prevalent in freshwater lakes.** Indicator linear regression analysis (*R*^2^ = 0.82, *F*_86,8_ = 133, *P* < 0.001, *n* = 10) was based on the relative recovery of dormant OTUs across a range of cutoffs (0.1–0.9) calculated as 1 - (rRNA recovery/rDNA recovery) for each OTU from rDNA and rRNA community libraries. Dormancy was 16% lower in hypersaline than freshwater lakes measured as the percent decrease between the significantly different *y*-intercepts (*P* < 0.001) from the equations for each lake.

Dormant bacteria were detected in hypersaline and freshwater lakes and dormancy was more prevalent in freshwater than extreme hypersaline environments. Based on indicator variable multiple regression results, the effect of lake type on the recovery of dormant OTUs was reflected in a difference between the *y*-intercepts in the equations for each lake type (*R*^2^ = 0.82, *F*_86,8_ = 133, *P* < 0.001, Eqs 1 and 2, **Figure 3**). Specifically, the percentage of bacterial recovery classified as dormant was 16% lower (percent decrease based on *y*-intercepts) in hypersaline than freshwater lakes across a wide range of cutoffs.

(1) Freshwater⁢ : % re⁢covery⁢ of⁢ dormant⁢ OTUs=80.4−59.2(cutoff)⁢

(2) Hypersaline⁢ : % re⁢covery⁢ of⁢ dormant⁢ OTUs=67.6−62.4 (cutoff)⁢

In general, the recovery of bacteria classified as being dormant decreased linearly as the cutoff estimating dormancy increased, and there were no interactions between the slopes and intercepts, suggesting that our conclusions were robust across the entire range of cutoffs. Alternatively, the effect of lake type on the number of dormant OTUs was similar leading to the overall model:

(3) % Dormant⁢ OTUs=74.0−60.8⁢ (cutoff)⁢

(*R*^2^ = 0.68, *F*_86,8_ = 186, *P* < 0.001, Supplementary Figure S2). As the cutoff increased or became more stringent, the number of OTUs classified as dormant decreased with values ranging from 59.3% ± 6.92 to 39.6% ± 1.22.

### Environmental Drivers of Bacterial Dormancy

**FIGURE 4:**
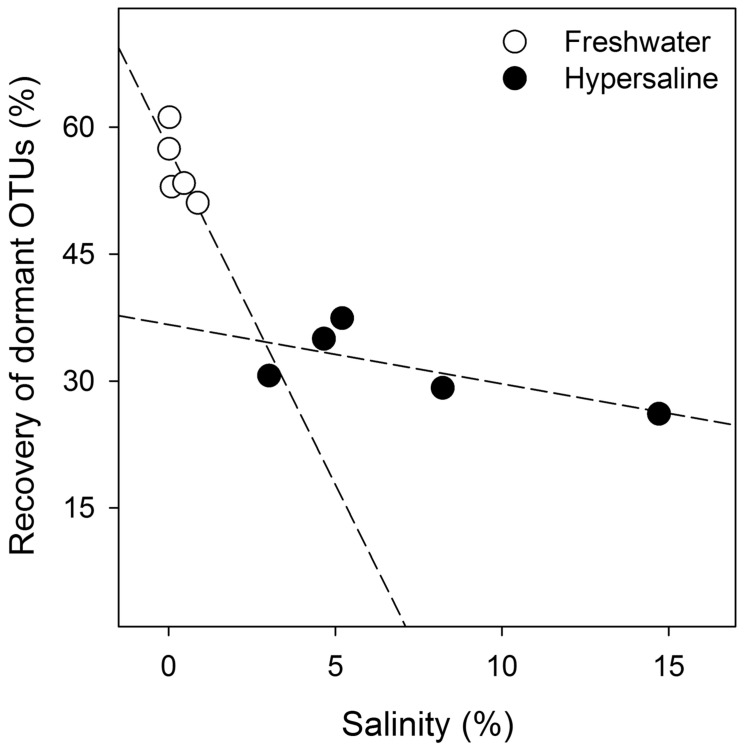
**Bacterial dormancy decreases as lake salinity increases.** The indicator regression analysis (*R*^2^ = 0.96, *F*_8,1_ = 50.0, *P* < 0.001, *n* = 10) was based on the relative recovery of dormant OTUs at the cutoff of 0.5 from the equation 1 - (rRNA recovery/rDNA recovery). Dormancy was calculated for each OTU from rDNA and rRNA community libraries.

Salinity influenced dormancy in both hypersaline and freshwater lakes. The multiple regression model that best predicted the relative recovery of dormant OTUs differed by lake type for salinity (Eqs 4 and 5; *P* < 0.05) but also included TP to a lesser extent (*P* < 0.09, *R*^2^ = 0.96, *F*_8,1_ = 50.0, *P* < 0.001; **Figure 4**). Of all possible models, this one generated the lowest AIC score (54) with a ΔAIC of 4.4, and resulted in the following equations:

(4) Hypersaline⁢ : % re⁢covery⁢ of⁢ dormant⁢ OTUs=57.6−9.10⁢ (salinity⁢ %)+0.04⁢ (TP)⁢

(5) Freshwater⁢ : % re⁢covery⁢ of⁢ dormant⁢ OTUs=33.0−0.58⁢ (salinity⁢ %)+0.04⁢ (TP)⁢

Dormancy decreased with increasing salinity across both lake types, but the effect of salinity on dormancy was more pronounced in freshwater systems where the slope describing salinity’s impact on dormancy was 16-times higher in freshwater than hypersaline lakes, substantially contributing to a 43% decline in dormancy (percent decrease based on y-intercepts, *P* < 0.001) in freshwater to hypersaline lakes. Dissolved oxygen, pH, and TN did not significantly influence the relative recovery of OTUs classified as dormant and were not included the models. We were unable to identify a model that linked environmental drivers and the number of dormant OTUs.

### Relationship between Commonness, Rarity, and Dormancy

**FIGURE 5:**
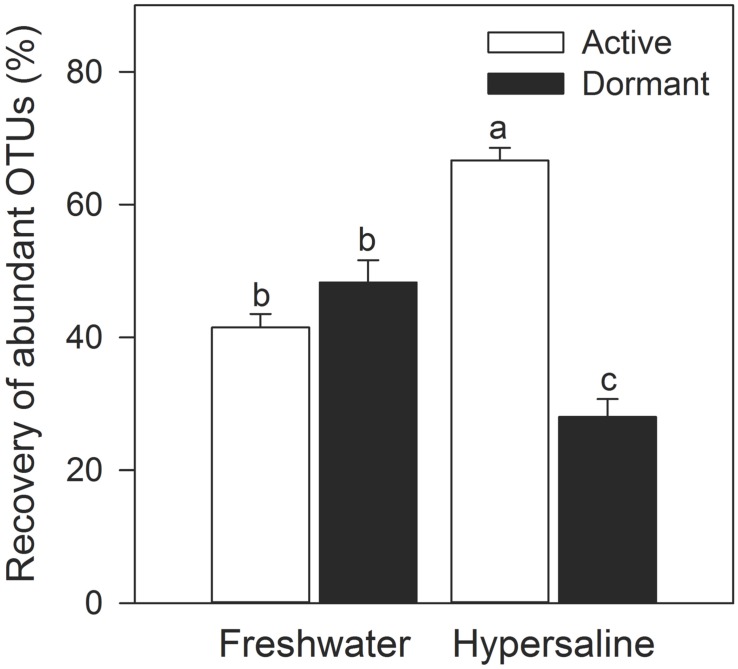
**Abundant bacteria were more likely to be active than dormant in hypersaline lakes.** OTUs with a relative recovery ≤ 0.1% were considered rare, while OTUs with a relative recovery > 0.1 were considered abundant based on rDNA community libraries (97% similarity cutoff). Values are means ± SEM (*n* = 5) with different letters indicating significant differences (*P* < 0.05) based on a two-way ANOVA and a Tukey’s HSD test.

A greater percentage of abundant bacteria were classified as active rather than dormant in hypersaline lakes. Specifically, the recovery of abundant OTUs (>0.1% relative recovery) comprised the majority of rDNA in extreme communities (95% ± 1.1) with 2.4-times more of this recovery being dormant than active (two-way ANOVA, lake × active vs. dormant, *F* = 78.1, *P* < 0.0001, df = 1, **Figure 5**). Further, the recovery of abundant and active taxa was 61% higher in extreme than freshwater communities. The relative recovery of rare active and dormant OTUs were similar across lakes (two-way ANOVA lake × active vs. dormant, *F* = 0.32, *P* = 0.58, df = 1), with values ranging from 6.9% ± 2.5–1.7% ± 0.40.

**FIGURE 6:**
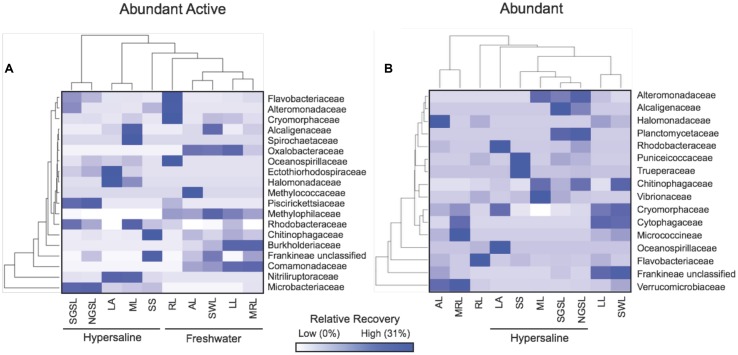
**Heat map showing the distribution of abundant active (A) and dormant (B) lake taxa in 16–19 bacterial families.** Values are based on means (*n* = 5) with hierarchal clustering of lakes (bottom) and families (left) that contributed ≥ 1% of the relative recovery to any rDNA lake community.

Between hypersaline and freshwater lakes, there were robust taxonomical differences in abundant active and dormant taxa. Differences in abundant and active bacteria between lake types were localized in five families within two phyla (i.e., Actinobacteria and Proteobacteria), which accounted for upward of 46% of the recovery in any lake (**Figure 6**). For example, the percentage of abundant and active OTUs in the Microbacteriaceae (Actinobacteria, hypersaline = 23% ± 7.5, freshwater = 0.86% ± 0.84), Nitriliruptoraceae (Actinobacteria, hypersaline = 13% ± 7.6, freshwater = 0%,), and Rhodobacteraceae (Alphaproteobacteria, hypersaline = 2.0% ± 0.68, freshwater = 0.49% ± 0.28) were at least 26-times higher in hypersaline than freshwater lakes, while Burkholderiaceae and Comamonadaceae (Betaproteobacteria) were absent in hypersaline lakes but accounted for 4.6% ± 2.1 and 12% ± 3.5 of the community in freshwater environments, respectively. Alternatively, taxonomical patterns among abundant and dormant taxa were apparent in freshwater systems where the recovery of families: Verrucomicrobiaceae (Verrucomicrobia, hypersaline = 0.20% ± 0.11, freshwater = 13% ± 6.0), Flavobacteriaceae (Bacteriodetes, hypersaline = 0.41% ± 0.33, freshwater = 6.1% ± 4.8), and an unclassified Frankineae family (Actinobacteria, hypersaline = 0.50% ± 0.40, freshwater = 8.5% ± 3.9) were at least 14-times higher in freshwater than hypersaline lakes.

## Discussion

Bacterial dormancy is often assumed to be a survival mechanism allowing taxa to contend with harsher environmental conditions (Stevenson, 1978; Nicholson et al., 2000; Dworkin and Shah, 2010; Lennon and Jones, 2011). However, in this study, we found bacterial dormancy to be less common in extreme hypersaline habitats than seemingly more benign freshwater habitats. Bacteria in hypersaline lakes were classified as dormant at a similar percentage as freshwater bacteria, and the proportion of the community exhibiting dormancy was 16% lower in hypersaline than freshwater lakes across a range of cutoffs describing activity. In both lakes, our activity estimates were influenced by salinity and to a lesser extent phosphorus. Saltier conditions in both freshwater and hypersaline lakes increased bacterial activity within communities, suggesting that salinity is a robust environmental filter structuring activity. Our results suggest that highly adapted bacterial communities in extreme environments need dormancy less often to survive.

### Hypersaline Bacterial Communities

Hypersaline bacterial communities supported relatively low levels of bacterial diversity consisting of taxa from one dominant phylum and two Proteobacteria subclasses. Hypersaline environments are generated as waters containing high concentrations of salts flow into an endorheic lake and are concentrated as evaporation outputs exceed precipitation inputs (Ollivier et al., 1994; Boutaiba et al., 2011). Across these lakes, as with all extreme environments from acid seeps and deep-sea thermal vents to glacial ice and acid mine drainage, adverse abiotic conditions select for bacterial communities composed of extremotolerant bacteria and extremophiles (Baker and Banfield, 2003; Miroshnichenko and Bonch-Osmolovskaya, 2006; Seufferheld et al., 2008; Anesio and Laybourn-Parry, 2012). In hypersaline lakes, the primary adverse condition, hypersalinity and the osmotic stress that it induces, selects a subset of halophilic taxa from regional species pool (Wu et al., 2006; Wang et al., 2011; Logares et al., 2013; Tazi et al., 2014). We found evidence supporting this as hypersaline conditions selected for unique assemblages of bacteria that were compositionally distinct from freshwater communities. Our lakes contained a range of salinity from 3.0 to 15%, and, thus communities contained both “salt loving,” halophilic and halotolerant bacteria, which can exist in water up to 15% salinity (Pikuta et al., 2007). Specialized halophiles contributed to hypersaline communities that were 50% less diverse than their more benign analog with Cyanobacteria, Alphaproteobacteria, and Gammaproteobacteria contributing upward of 70% of the sequences. All three of these taxonomical groups are consistently dominant in other saline environments as well (Tourova et al., 2007; Jiang et al., 2010; Lefort and Gasol, 2013).

### Hypersaline Lakes Contain More Active Microbes

The prevalence of dormancy did not rise in extreme environments. Based on our approach using rRNA: rDNA ratios to classify the activity of bacterial taxa, we found that the proportion of the community exhibiting dormancy was 16% lower in hypersaline than freshwater lakes across a range of cutoffs, and species were classified as dormant at a similar percentage in hypersaline as freshwater lakes. More extreme saline conditions did not select for higher levels of dormancy. The reverse was actually true. As extreme environments became more hypersaline, a greater proportion of the community was active. An explanation for this result may stem from halophiles being highly adapted to hypersalinity for optimal metabolism and growth (Madigan and Marrs, 1997; Harrison et al., 2013). For example, hypersaline environments generally select for a wide range of metabolic diversity, such as oxygenic and anoxygenic phototrophs, obligate and facultative aerobic heterotrophs, fermenters, denitrifiers, sulfate reducers, and methanogens (Ollivier et al., 1994; Burke and Knott, 1997; Ciulla et al., 1997). However, as salinity continues to rise, metabolic diversity dramatically decreases (Oren, 2002; Pikuta et al., 2007). Thus, as hypersalinity intensifies, the resulting bacterial communities may become more specialized, perform fewer functions but remain predominately active.

### Salinity and P Drive Bacterial Dormancy

Saltier conditions in both freshwater and hypersaline lakes increased activity. A rise in salinity spanning less than a single percent (0.01–0.87%) across freshwater lakes corresponded to a 17% decrease in the recovery of dormant taxa exhibiting dormancy. Additionally, a five-fold increase in salinity (3.0–15%) among hypersaline lakes corresponded to a 30% decrease in the recovery of dormant taxa. Thus, salinity seemed to act as a strong environmental filter selecting for not only active extremophiles but also active freshwater bacteria able to contend with and thrive under saltier conditions. The immense effects of salinity on activity may be best explained or mirrored by the importance of salt concentrations to community composition. For example, across multiple biomes and in freshwater lakes, microbial community structure and diversity are primarily structured by salinity rather than temperature, pH, or other physical and chemical factors (Lozupone and Knight, 2007). As for hypersaline lakes, salinity levels exert immense selective pressure on bacterial species (Canganella and Wiegel, 2011). To remain active under saltier conditions, we expect most bacteria to cope with increasing or toxic levels of Na^+^ and potential desiccation stress by using osmoregulation resistance strategies (Oren, 2002, 2008; Canganella and Wiegel, 2011). This coping mechanism requires energy to actively export Na^+^ ions and synthesize and/or accumulate organic compatible solutes such as polyols, sugars, amino acids, and amines (Detkova and Boltyanskaya, 2007; Canganella and Wiegel, 2011). In light of these costs, it may be more beneficial/efficient for salt tolerant or salt loving taxa to just remain active and cope with osmotic stresses instead of entering a state of dormancy.

Phosphorus availability appeared to regulate bacterial activity even in extreme environments. Regardless of lake type, we found a trend (*P* < 0.09 marginally significant) where higher TP concentrations related to a lower percentage of the community estimated to be in a dormant state. Our results support previous evidence that low concentrations of TP influence dormancy in freshwater systems (Jones and Lennon, 2010). In freshwater lakes, selection pressures may drive bacteria to develop different patterns of resource use or functional traits allowing them to occupy different niches. Under low resource availability, dormancy confers a competitive advantage to bacteria as they avoid starvation and escape death. As nutrient levels decline, bacteria enter dormancy by forming cysts or endospores (Sussman and Douthit, 1973; Segev et al., 2012), creating persister cells (Rotem et al., 2010; Maisonneuve et al., 2011), or, simply suspending normal metabolic activity (Lennon and Jones, 2011). The role of competition is controversial in extreme environments and the impact of resources availability on activity remains unclear. However, there is a tendency for competition to decline as conditions become more stressful (Fišer et al., 2012) due to limitation in the number of niches for bacteria to occupy (Oren, 2002). In addition to salinity, we propose that competition for essential resources in extreme environments exerts some pressure on activity.

### Abundant Taxa Are Disproportionately Active

Communities are unevenly distributed with a few dominant species being numerically abundant and contributing overwhelmingly to the overall community, while rare taxa are thousands in number and contribute little in terms of abundance (Pedrós-Alió, 2012). However, overlaid on top of this seemingly universal shape to bacterial rank abundance curves lies the uncertainty of activity (Shade et al., 2014; Aanderud et al., 2015). We found the same ubiquitous rank abundance curve in both hypersaline and freshwater lakes, but hypersaline abundant taxa were disproportionately estimated as being active. These dominant and active taxa were localized in families adapted to a range of salinities. For example, Microbacteriaceae are predominantly aerobic, planktonic, and halotolerant bacteria with the potential to persist and thrive at multiple salinity levels (Han et al., 2003; Jiang et al., 2010; Jang et al., 2013), and Rhodobacteraceae of the Alhpaproteobacteria are halotolerant, moderately thermophilic chemoorganotrophs and photoheterotrophs common in water, biofilms, and microbial mats that withstand fluctuations in salinity (Denner et al., 2006; Farias et al., 2013; Lindemann et al., 2013). Abundant and active Nitriliruptoraceae are haloalkaliphic bacteria that require high salt levels to decompose organic C containing nitrile groups (Sorokin et al., 2009). The activity of abundant freshwater taxa may relate to differences in C source availability. For example, the Comamonadaceae, which were abundant and active in freshwater lakes, are associated with the decomposition of cyanobacteria biomass, particularly *Microcystis* species, and wastewater streams (Li et al., 2012; Krustok et al., 2015). Alternatively, Verrucomicrobiaceae and Flavobacteriaceae, which were abundant but dormant in freshwater lakes, are associated with high levels of laminarin and xylan from algal sources. Collectively, Verrucomicrobiaceae and Flavobacteriaceae produce six endo-acting polysaccharide hydrolases facilitating the decomposition of polysaccharides and cell wall constituents (Cardman et al., 2014). Unfortunately, we did not measure C sources in our water samples. Our results suggest that dormancy is unnecessary for taxa to achieve dominance in extreme conditions.

### Reasonable and Robust Dormancy Estimates

Our estimates of dormancy represent an approximation of bacterial activity with the cutoffs separating active from dormant taxa conserved over a wide range of values. We classified taxa as either active or dormant bacteria based on rRNA: rDNA ratios using the recovery of individual taxa in active (rRNA) and total (rDNA) bacterial communities. Inferring activity based on rRNA is a commonly applied approach to characterize growing or active bacteria (Campbell and Kirchman, 2013; Hugoni et al., 2013); however, rRNA alone may not be a reliable indicator of the metabolic state of a bacterium (Blazewicz et al., 2013). In the case of dormancy, inactive bacteria may contain measureable amounts of rRNA, and in specific cases, specialized cells (e.g., akinetes of some Cyanobacteria), may contain more rRNA in an inactive than active state (Sukenik et al., 2012). By estimating bacterial dormancy for each taxa independently using rRNA: rDNA ratios, we compensated for potential taxonomic discrepancies associated with rRNA and activity (Blazewicz et al., 2013). Our results are based on a rRNA: rDNA ratio cutoff of ≥0.5. But even if the cutoff was stricter, where the total recovery of an OTU (active and inactive cells) was at least 10-times the recovery of RNA transcripts being produced (cutoff = 0.9), our major findings were the same. We do concede that our approach does not perfectly discriminate between dormant and extremely slow-growing bacteria populations (Jones and Lennon, 2010). But slow-growers and dormant individuals may respond, grow, and resuscitate similarly following changes in environmental cues (Kjelleberg et al., 1987; Choi et al., 1999). Thus, we feel our estimate is an appropriate metric quantifying the baseline effects of dominant ecosystem characteristics on activity.

## Conclusion

Halophilic and halotolerant bacteria may employ dormancy to facilitate their long-term persistence and maintain bacterial diversity in extreme environments, but a lower proportion of these communities appear to be dormant. The overarching extreme condition (i.e., hypersalinity) not only structured the less diverse and distinct bacterial communities, but also activity levels. To further justify our conclusion, shifts in dormancy need to be investigated as members of extreme communities are pushed beyond their optimal conditions. In general, the prevalence of dormancy is expected to rise as a species moves away from its niche (Lennon and Jones, 2011). In extreme environments, the primary condition defining the environment (e.g., salinity, acidity, and temperature) vary both seasonal or episodically in intensity (Ferris et al., 2003; Detkova and Boltyanskaya, 2007; Yücel et al., 2013). If dormancy is evaluated through time as these primary and other factors fluctuate, we will be able to better identify the extent of dormancy and the cues governing activity in extreme environments.

## Author Contributions

ZA, JV, DB, and AH designed the study. ZA, JV, and TM conducted the experiments. ZA, JV, JL, TM, DB, and AH analyzed and interpreted the data, ZA, JV, JL, TM, DB, and AH helped write and review the manuscript. ZA agrees to be accountable for all aspects of the work in ensuring that questions related to the accuracy or integrity of any part of the work are appropriately investigated and resolved.

## Conflict of Interest Statement

The authors declare that the research was conducted in the absence of any commercial or financial relationships that could be construed as a potential conflict of interest.

## Footnotes

1 http://dnasc.byu.edu
