# Supplementary material for: Bacterial Dormancy Is More Prevalent in Freshwater than Hypersaline Lakes
Source: Front Microbiol. 2016 Jun 9;7:853. doi: 10.3389/fmicb.2016.00853 (PMC4899617; doi:10.3389/fmicb.2016.00853)
Supplement: Supplementary file 1 [file Table_1.PDF]

SUPPLEMENTARY TABLE 1 | Characteristics and locations of hypersaline and freshwater lakes (NA = not available).

	Elevation (m.a.s.l.)	Surface area (km^2)	Mean depth (m)	Location
Hypersaline				
Great Salt Lake, North Arm, UT	1285	2175	3.9	N 41˚25.944' W 112˚39.777'
Great Salt Lake, South Arm, UT	1280	2228	4.3	N 41˚03.641' W 112˚14.668'
Salton Sea, CA	-77	974	9.5	N 33˚26.591' W 115˚50.952'
Lake Abert, OR	1287	148	2.1	N 42˚37.554' W 120˚11.191'
Mono Lake, CA	1948	180	17	N 37˚56.663' W 119˚01.048'
Freshwater				
Mormon Lake, ID	1542	11	2.4	N 43˚16.007' W 114˚50.181'
Riffe Lake, WA	249	48	NA	N 46˚29.261' W 122˚11.228'
Arivaca Lake, AZ	1153	0.36	9.3	N 31˚31.802' W 111˚15.137'
Lily Lake, CO	2728	0.17	6.4	N 40˚18.477' W 105˚32.521'
Silverwood Lake, CA	1020	4.0	6.1	N 34˚17.122' W 117˚20.523'

SUPPLEMENTARY TABLE 2 | Chemistry of individual freshwater and hypersaline lakes. Data are values from a composite water sample consisting of three subsamples.

Lake	Dissolved O_2 (µmol L^{-1})	Electrical conductivity (dS m^{-1})	pH	Salinity (%)	Temperature (˚C)	Total N (µmol L^{-1})	Total P (µmol L^{-1})
Freshwater Lakes							
Arivaca Lake	232	0.19	7.11	0.01	21.3	63.2	1.15
Lily Lake	201	1.3	6.56	0.08	14.9	21.2	0.066
Mormon Lake	220	13	6.91	0.87	18.2	31.4	28.0
Riffe Lake	289	0.39	6.83	0.02	14.5	9.21	0.127
Silverwood Lake	222	7.6	7.61	0.46	20.8	28.1	3.50
Hypersaline Lakes							
Albert Lake	151	65	9.83	5.2	17.6	81.8	138
Great Salt Lake North Arm	178	155	7.49	15	18.7	353	53.5
Great Salt Lake South Arm	194	97	8.24	8.2	18.4	58.3	1.58

Mono Lake	156	60	9.81	4.7	18.7	93.5	158
Salton Sea	193	50	8.17	3.0	28.9	38.5	0.365
