# Supplementary figures and images for: Bacterial Dormancy Is More Prevalent in Freshwater than Hypersaline Lakes
Source: Front Microbiol. 2016 Jun 9;7:853. doi: 10.3389/fmicb.2016.00853 (PMC4899617; doi:10.3389/fmicb.2016.00853)

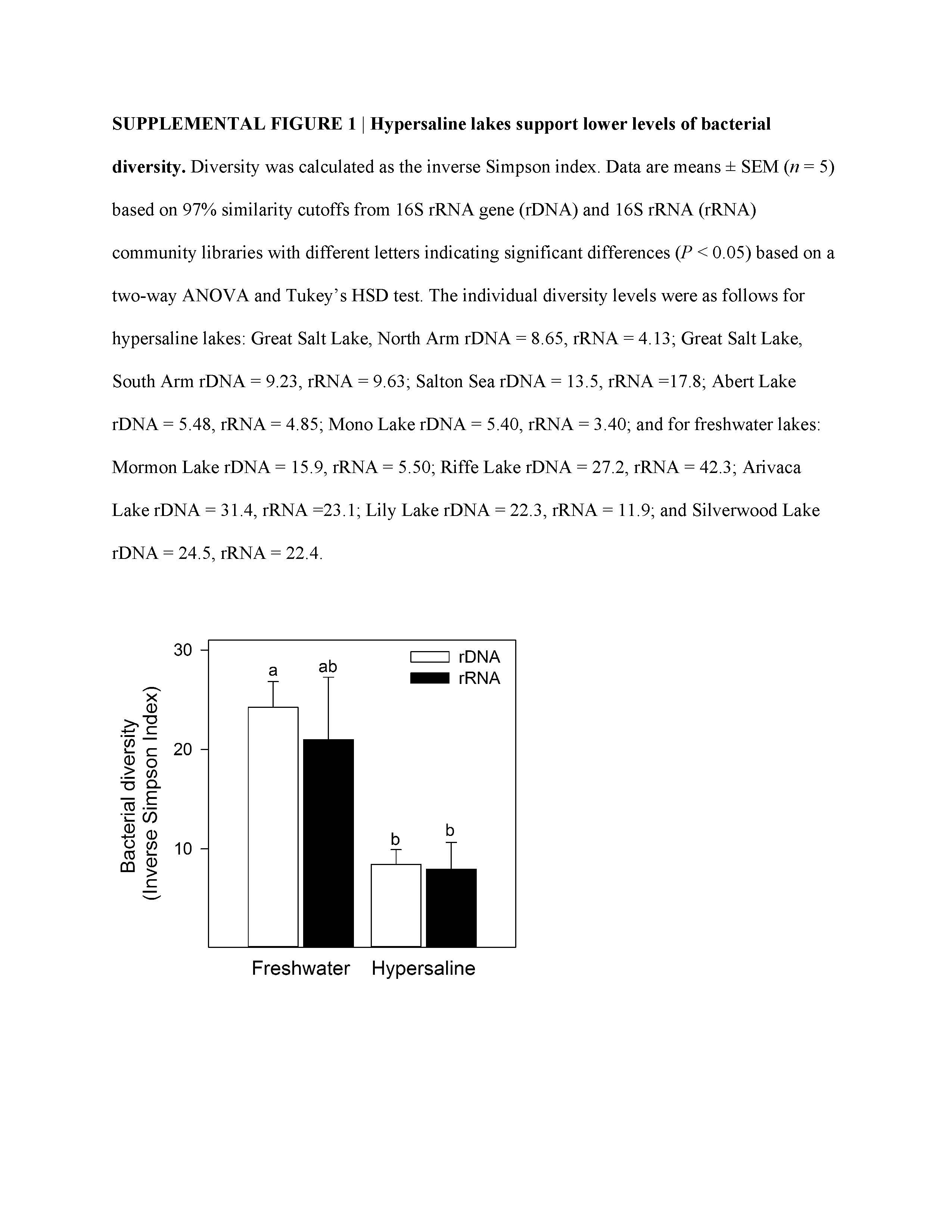

Supplement: Supplementary file 2 [file Image_1.JPEG]

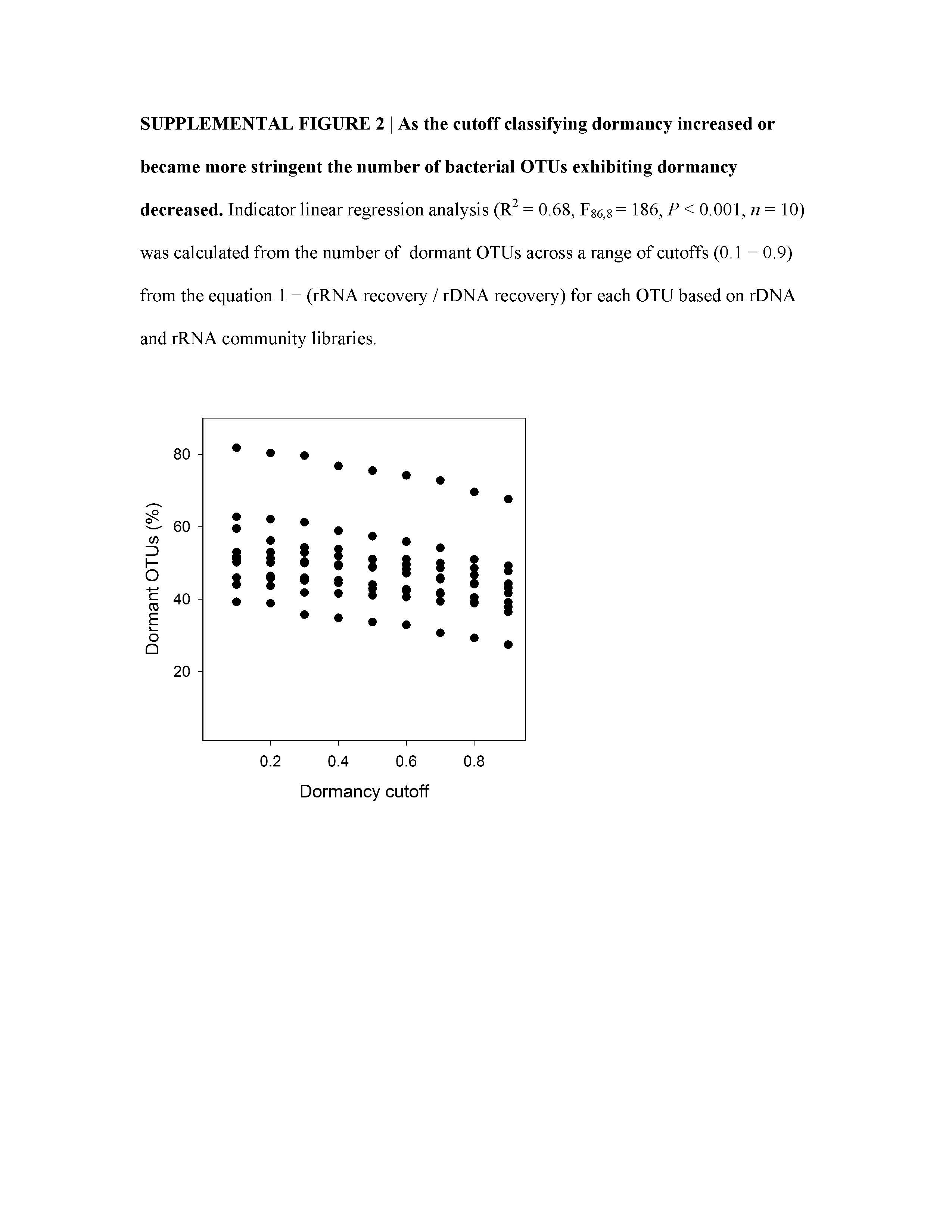

Supplement: Supplementary file 3 [file Image_2.JPEG]
